# Serum glial fibrillary acidic protein is elevated in early-stage late-onset essential tremor and associated with tremor progression

**DOI:** 10.1007/s00702-025-02970-8

**Published:** 2025-06-15

**Authors:** Lukas Gattermeyer-Kell, Michael Khalil, Daniela Kern, Sebastian Franthal, Petra Katschnig-Winter, Mariella Kögl, Rina Demjaha, Cansu Tafrali, Edith Hofer, Reinhold Schmidt, Christian Enzinger, Petra Schwingenschuh

**Affiliations:** 1 Department of Neurology, Medical University of Graz, Auenbruggerplatz 22, 8036 Graz, Austria; 2 Institute for Medical Informatics, Statistics and Documentation, Medical University of Graz, Graz, Austria

**Keywords:** Essential tremor, Tremor, Serum glial fibrillary acidic protein, GFAP, Biomarker

## Abstract

The role of neurodegeneration in essential tremor (ET) remains debated, particularly in patients with late disease onset. Neuropathological studies have identified structural changes in the Purkinje cells and its connections. Recent studies additionally suggested a role of cerebellar astrocytes. Increased levels of serum glial fibrillary acidic protein (sGFAP), an astrocytic intermediate filament, were found in various neuroinflammatory and neurodegenerative diseases. The objective of this case–control study was to investigate the role of sGFAP in ET focusing on early-stage late-onset patients. sGFAP was quantified by single molecule array at baseline and follow-up in 36 ET-patients (median follow-up period 5.3 years) and 36 age-matched healthy controls (4.9 years). ET-patients were assessed both at baseline and follow-up using the Fahn–Tolosa–Marin–Tremor-Rating-Scale. The ET group was stratified (1) by median age at onset and median disease duration in early-stage late-onset and early-onset/late-stage ET, and (2) by median sGFAP-level at baseline. Early-stage late-onset ET-patients had higher baseline-sGFAP than controls (p = 0.023) and higher follow-up-sGFAP and annual sGFAP-increase than both controls (p = 0.023; p = 0.007) and early-onset/late-stage ET-patients (p = 0.021; p = 0.024). Baseline sGFAP-level correlated with tremor severity at follow-up in the early-stage late-onset (r_s_ = 0.704, p = 0.011) but not in the early-onset/late-stage group. Patients with high compared to low sGFAP-baseline levels had later disease onset (p < 0.001) and sGFAP-increase was associated to tremor progression only in high sGFAP-patients (p = 0.041). ET-plus and pure-ET-patients did not differ in any of the sGFAP-parameters. sGFAP is elevated in early stages of late-onset ET and associated to tremor progression, substantiating the role of a neurodegenerative process in ET in this subgroup.

## Introduction

Essential tremor (ET) has been increasingly recognized as a heterogenous syndrome (Louis 2018; Hopfner and Deuschl 2018), characterized by variable presence of other motor and non-motor symptoms (Louis 2018). To overcome this heterogeneity and give insight into possible underlying pathophysiologic processes, ET-subtypes based on different traits have been suggested. The concept of ET-plus is based on the presence of additional clinical signs (Bhatia et al. 2017). Other authors have suggested subtyping ET based on age at tremor onset, differentiating early versus late-onset (or aging-related) ET (Hopfner et al. 2016; Deuschl et al. 2015), with the latter showing faster tremor progression and being suggestive of a neurodegenerative process (Hopfner et al. 2016). While there is considerable post-mortem evidence of neurodegeneration in ET (Louis and Faust 2020; Louis et al. 2023), in-vivo findings are growing yet still limited (Louis et al. 2024; Salinas et al. 2024; Franthal et al. 2023; Mostile et al. 2021), with involvement of the cerebellum as the most consistent finding in neuroimaging studies (Holtbernd and Shah 2021).

Simultaneously, the field of neurology has seen a surge in biomarker research (Lleó 2021). In this respect, (serum) neurofilament light chain (sNfL), a biomarker of neuroaxonal damage (Khalil et al. 2024, 2018), was found elevated in various neurological diseases (Wang et al. 2019; Huang et al. 2022) including ET (Franthal et al. 2023; Salinas et al. 2024; Louis et al. 2024). A recent study showed that short disease duration ET-patients had a higher sNfL-increase during a 5-years observational period than long disease duration ET-patients, and sNfL-increase was associated to tremor progression, hinting towards a neurodegenerative process happening in early disease (Franthal et al. 2023).

Recently, blood glial fibrillary acidic protein (GFAP), an astrocytic intermediate filament, has been shown to be elevated in various neuroinflammatory and neurodegenerative diseases (Abdelhak et al. 2022). While neuropathological research in ET has, as yet, primarily focused upon Purkinje cell alterations (Louis 2015; Faust et al. 2024), a recent study found changes in terminal structures of Bergmann glia in ET, suggesting a possible pathophysiological role of these astrocytes (Ruff et al. 2023). Moreover, differential expression of astrocytic excitatory amino-acid-transporter-2 in the dentate nucleus and cerebellar cortex has been speculated to play a modulating role in possible excitotoxicity in ET-pathophysiology (Wang et al. 2016).

Against this background, the aim of this exploratory pilot study was to compare serum GFAP (sGFAP) in ET-patients to healthy controls (HC). Considering the probable role of neurodegeneration, especially in late-onset ET, and the finding of high sNfL-increase in short disease duration ET-patients, we primarily focused on late-onset ET-patients in early disease stages, aiming to determine whether an approach implementing blood-based biomarkers substantiates this neurodegenerative disease model in putative clinical ET-subtypes. Moreover, we aimed to explore the relationships of sGFAP-levels and tremor severity.

## Methods

### Participants

Patients were consecutively recruited from an ongoing single-center registry study on movement disorders (Prospective Movement Disorders Registry Graz, PROMOVE) at the Medical University of Graz, Austria, starting in 2010 up until 2021. Details of the study have been published before (Franthal et al. 2023). For this study, we included all 36 patients above the age of 18 and a clinical diagnosis of ET according to the current MDS consensus statement (Bhatia et al. 2017), a baseline Mini-Mental State Examination > 26, and available baseline blood samples. A baseline Mini-Mental State Examination of > 26 points was required for patients to be eligible to sign the informed consent form at initial study inclusion. The majority of patients with ET (22/36) underwent single‐photon emission computed tomography imaging with DaTSCAN™ (Ioflupane 123‐I SPECT), which was reported as normal in all of them. DaTSCAN™ was a facultative part of the study, and the remaining 14/36 patients chose not to undergo the examination without having to state specific reasons.

All ET-patients underwent clinical assessment in medication-off-state including Fahn–Tolosa–Marin Tremor Rating Scale (FTM) (Fahn et al. 1988) at baseline and follow-up. Annual change in FTM (in points) was calculated by dividing the absolute change in FTM by the individual duration of the observational period in years. ET patients were classified as pure-ET or ET-plus predominantly retrospectively based on videotaping of the baseline visit; soft signs considered in this group were resting tremor, questionable dystonia, and impaired tandem gait.

36 age-, sex- and body-mass-index-(BMI)-matched HC without first-degree relatives with any movement disorder were recruited from an ongoing community-dwelling aging cohort (Schmidt et al. 2003).

Patients and controls were not included with a history or symptoms of dementia, head trauma, epilepsy, stroke, or brain surgery. All ET-patients and HC underwent 3 T magnetic resonance imaging scans of the brain to exclude relevant structural brain abnormalities including chronic vascular encephalopathy and brain atrophy.

29/36 (80.6%) patients and 32/36 (88.9%) HC had a follow-up visit after a median [interquartile range] of 5.3 [3.1–5.8] years and 4.9 [4.5–6.1] years, respectively.

ET patients were stratified into two subgroups according to median age at onset (53.4 [41.2–62.7] years) and median disease duration at baseline (10.7 [4.8–21.4] years): Patients with both a disease duration below median and an age at onset above median were grouped as the early-stage-late-onset-(ESLO)-ET-subtype (baseline n = 15, follow-up n = 12), whereas patients with a long disease duration or an early disease onset were termed as the late-stage/early-onset-(LSEO)-ET-subtype (baseline n = 21, follow-up n = 17). A mathematically based median split was chosen with respect to the lack of generally accepted cut-offs for early vs. late-onset ET or short vs. long disease duration, respectively.

To explore potential differences in ET-patients with high or low sGFAP-levels including motor symptom progression, we stratified the total ET group (independently of age at onset or disease duration) into high-sGFAP-patients with an sGFAP-level at baseline above median (124.2 [84.1–173.3] pg/mL; n = 18) and low-sGFAP-patients with an sGFAP-level below median (n = 18) (Kuhle et al. 2024).

### Standard protocols approvals, registrations, and patient consents

Approval was obtained from the institutional review board of the Medical University of Graz (21-345 ex 09/10). All participants gave written informed consent before inclusion in the study. This study conforms with the World Medical Association Declaration of Helsinki.

This observational study followed the Strengthening the Reporting of Observational Studies in Epidemiology (STROBE) checklist guidelines for case–control studies.

### Serum glial fibrillary acidic protein

Blood samples were obtained by peripheral venipuncture of all participants at baseline and follow-up. The specimens were centrifuged and frozen at −  80 °C following a standardized procedure (Teunissen et al. 2009). sGFAP-levels were measured with the Simoa^®^ GFAP single-plex discovery kit (reference number: 102336) on the HD-X platform (Quanterix, Billerica, MA, USA). The assays’ lower limit of quantification lies at 0.686 pg/mL and the limit of detection lies at 0.211 pg/mL. To ensure inter-assay precision all measurements were performed in duplicates; results with a coefficient of variation over 20% were excluded from our analyses. Annual change in sGFAP was calculated by dividing the absolute change in sGFAP in pg/mL by the individual duration of the observational period in years.

### Statistical methods

**Fig. 1:**
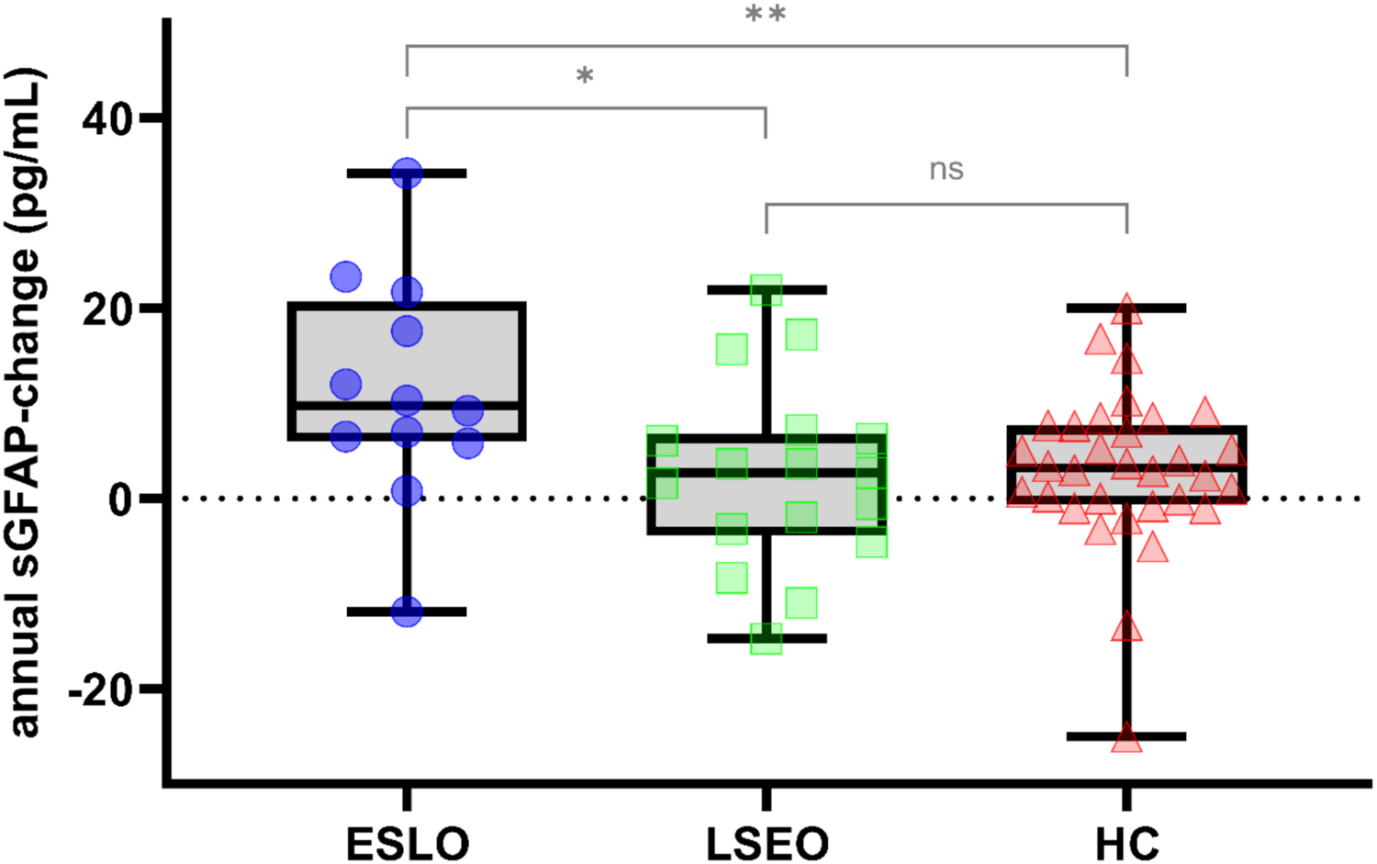
Annual change in sGFAP-level (pg/mL) in ESLO-ET (n = 12), LSEO-ET (n = 17) and HC (n = 32), respectively. p-values less than 0.05 are flagged with one asterisk (*), p-values less than 0.01 with two asterisks (**). *GFAP* serum glial fibrillary acidic protein, *ESLO* early-stage-late-onset, *ET* essential tremor, *LSEO* late-stage/early-onset,* HC* healthy controls, *ns* non-significant

**Fig. 2:**
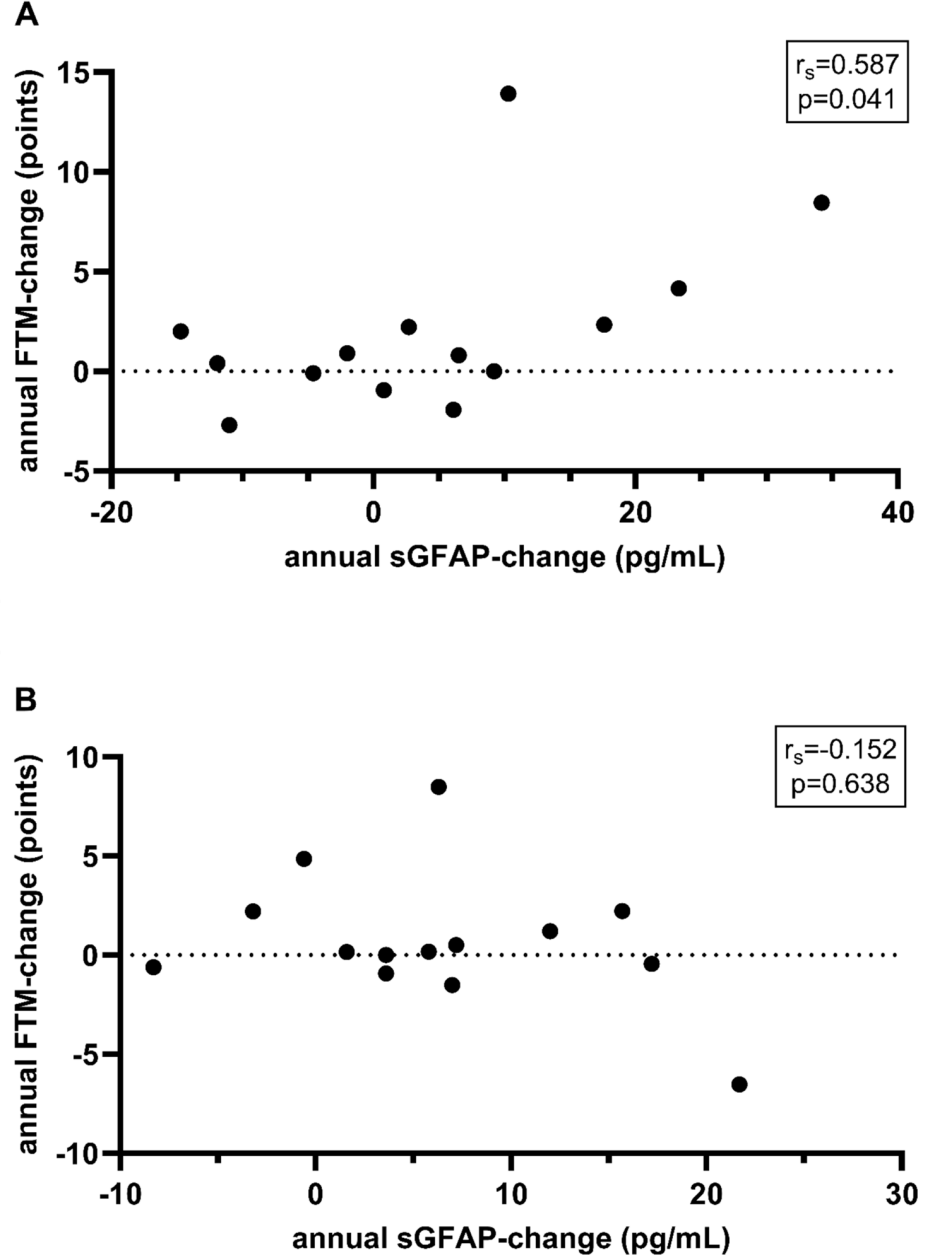
**A** Significant positive Spearman correlation (r_s_) between annual change in sGFAP-level (pg/mL) and annual change in FTM-score (points) in ET-patients with baseline sGFAP-level above median (n = 14) but **B** not in ET-patients with baseline sGFAP-level below median (n = 14). *sGFAP* serum glial fibrillary acidic protein, *FTM* Fahn–Tolosa–Marin–Tremor-Rating-Scale, *ET* essential tremor

Statistical analysis was performed using the software SPSS Statistics (IBM Statistics for Macintosh, Version 29). Data was summarized using descriptive statistics. Categorical variables are reported as absolute or relative frequencies. Continuous data are expressed as mean and standard deviation (mean ± SD) or median and interquartile range (median [IQR]), respectively. Assumptions of normal distributions were tested with the Shapiro–Wilk test. Group differences were calculated with χ^2^ test (categorical data), independent t-test (parametric data), or Mann–Whitney U test (non-parametric data), as appropriate. For subgroup analyses, only non-parametric tests were used due to the small sample size. Correlations analyses in these subgroups were performed using Spearman’s rank correlation coefficient. In correlation analyses multiple testing was accounted for by using the Benjamini–Hochberg procedure with a false discovery control level of α = 0.05. All p-values of < 0.05 were considered statistically significant. Figures 1 and 2 were generated using GraphPad Prism (GraphPad Prism Version 10.3.1 for Windows, GraphPad Software, Boston, Massachusetts USA, www.graphad.com).

## Results

### Total ET-patients vs. HC

Total ET-patients and HC did not differ in baseline age (p = 0.954), BMI (p = 0.839) or sex (p = 1.000; Table 1). While the median baseline sGFAP-level was numerically higher in ET-patients (124.2 pg/mL [84.1–173.3]) compared to HC (113.0 pg/mL [76.2–142.1]), this was not statistically significant (p = 0.156). Annual sGFAP-increase was numerically almost twice as high in ET-patients compared to HC (6.1 [− 1.3 to 13.8] vs. 3.2 [− 0.6 to 7.7] pg/mL), although this was not significant either (p = 0.292).

**Table 1:** Clinical and demographic data

	Total ET	ESLO-ET	LSEO-ET	HC
Total number at baseline, n	36	15	21	36
Number of participants with a follow-up visit, n	29	12	17	32
Follow-up period (years)	5.3 [3.1–5.8]	5.5 [1.7–5.8]	5.2 [3.6–5.8]	4.9 [4.5–6.1]
Age at baseline (years)	65.3 ± 6.6 (mean ± SD)	67.1 [64.2–70.5]	64.2 [56.1–71.2]	65.4 ± 7.3 (mean ± SD)
Sex, n (male/female) [% female]	19/17 [47.2]	9/6 [40.0]	10/11 [52.4]	19/17 [47.2]
Body mass index (kg/m^2^)	27.1 [24.3–30.0]	28.7 [26.5–30.0]	26.1 [23.3–30.3]	27.2 [24.2–31.2]
Disease duration at baseline (years)	10.7 [4.8–21.4]	4.9 [3.6–6.8]	20.4 [12.4–40.7]	–
Age at disease onset (years)	53.4 [41.2–62.7]	62.8 [58.6–64.9]	49.5 [18.0–52.3]	–
FTM at baseline (points)	29.5 [16.0–34.0]	19.0 [13.0–29.0]	33.0 [21.0–39.0]	–
FTM at follow-up (points)	27.5 [17.5–44.8]	25.0 [17.5–31.5]	34.5 [16.8–46.8]	–
Annual change in FTM (points)	0.5 [− 0.6 to 2.2]	0.6 [− 0.7 to 3.7]	0.3 [− 0.6 to 2.2]	–
MMSE at baseline (points)	29.0 [28.0–30.0]	29.0 [27.0–29.0]	29.0 [28.0–30.0]	29.0 [28.0–29.0]

Data are presented as median [interquartile range], unless otherwise specified

*ET* essential tremor, *ESLO* early-stage-late-onset, *LSEO* late-stage/early-onset, *HC* healthy controls, *SD* standard deviation, *FTM* Fahn-Tolosa-Marin Tremor Rating Scale, *MMSE* Mini Mental State examination

### ESLO vs. LSEO vs. HC

When comparing ESLO-patients to HC, the groups did not differ in age (p = 0.408), BMI (p = 0.664) or sex (p = 0.637; Table 1). ESLO-patients had a significantly higher sGFAP at baseline (137.7 [119.8–181.1] vs. 113.0 [76.2–142.1] pg/mL, p = 0.023) and follow-up (172.5 [134.6–200.8] vs. 142.4 [91.4–166.1] pg/mL, p = 0.023) and a higher annual sGFAP-increase (9.7 [6.0–20.6] vs. 3.2 [− 0.6 to 7.7] pg/mL, p = 0.007; Fig. 1) than HC.

While ESLO-patients and LSEO-patients did not differ in age (p = 0.191), BMI (p = 0.238), sex (p = 0.463; Table 1), or baseline sGFAP-level (137.7 [119.8–181.1] vs. 111.6 [78.2–156.1] pg/mL, p = 0.117), ESLO-patients had significantly higher follow-up sGFAP-levels (172.5 [134.6–200.8] vs. 114.0 [88.9–157.6] pg/mL, p = 0.021) and annual sGFAP-increase (9.7 [6.0–20.6] vs. 2.7 [− 3.9 to 6.7] pg/mL, p = 0.024; Fig. 1) than LSEO-patients.

LSEO-patients and HC, conversely, did not differ in age (p = 0.508), BMI (p = 0.519), sex (p = 0.707), sGFAP at baseline (p = 0.791) or follow-up (p = 0.462) nor annual sGFAP-increase (p = 0.571; Fig. 1).

Higher baseline sGFAP correlated significantly with higher follow-up FTM-scores (r_s_ = 0.704, p = 0.011) in the ESLO-group but not the LSEO-group (r_s_ = − 0.050, p = 0.854), while there was a significant positive correlation between baseline sGFAP and baseline age in all groups including HC (ESLO: r_s_ = 0.686, p = 0.005; LSEO: r_s_ = 0.582, p = 0.006; HC: r_s_ = 0.360, p = 0.031).

### High vs. low sGFAP

When comparing high to low sGFAP-patients, the high sGFAP-group was older both at baseline (median age 70.0 [67.6–73.9] vs. 60.7 [55.5–64.4] years, p < 0.001) and disease onset (median age at onset 62.6 [53.7–64.9] vs. 49.9 [16.6–53.2] years, p < 0.001). Age did not correlate with any of the parameters of interest (baseline sGFAP, annual sGFAP-increase, baseline/follow-up FTM, annual FTM-increase), neither within the high nor the low sGFAP-group (false discovery rate (FDR)-corrected p for all > 0.05). However, only in the high sGFAP-group, higher baseline sGFAP-levels were associated with higher FTM-scores both at baseline (r_s_ = 0.534, FDR-corrected p = 0.033) and follow-up (r_s_ = 0.761, FDR-corrected p = 0.006). High sGFAP-patients with low baseline FTM-scores (i.e., early-stage patients) had a higher annual sGFAP-increase (r_s_ = −0.803, FDR-corrected p = 0.002). Furthermore, annual sGFAP-increase significantly correlated with annual FTM-increase (r_s_ = 0.587, FDR-corrected p = 0.041; Fig. 2A), i.e., tremor progression, only in the high sGFAP-group, while none of these correlations was present in the low sGFAP-group (Fig. 2B). Further investigating this correlation in the high sGFAP-group revealed that annual sGFAP-increase correlated significantly with both annual increase of the FTM subscores A (r_s_ = 0.587, p = 0.027) and B (r_s_ = 0.543, p = 0.045) but not C (r_s_ = 0.411, p = 0.144).

### Presence of additional symptoms

16/36 (44.4%) patients were retrospectively classified as ET-plus, while the remaining 20/36 (55.6%) were categorized as pure-ET; ET-plus and pure-ET-patients did not differ in median baseline (128.9 [95.6–173.2] vs. 124.0 [83.4–174.1] pg/mL, p = 0.789) or follow-up sGFAP-levels (132.3 [121.6–165.4] vs. 146.9 [108.8–194.8, p = 0.914) nor annual sGFAP-increase (7.0 [− 2.0 to 15.7] vs. 6.0 [1.2–11.1], p = 0.948). ESLO- and LSEO-groups did not differ in their proportion of ET-plus-patients (6/15 (40.0%) vs. 10/21 (47.6%), p = 0.65).

In addition, patients with head tremor (n = 6 at baseline, n = 5 at follow-up) and patients without head tremor (n = 30 at baseline, n = 24 at follow-up) did not differ in sGFAP-levels at baseline (155.5 [79.9–221.0] vs. 119.9 [89.7–167.0] pg/mL, p = 0.520) or follow-up (128.6 [121.6–189.1] vs. 146.9 [108.8–178.1] pg/mL, p = 1.0) nor annual change in sGFAP-levels (7.0 [− 4.6 to 7.2] vs. 6.0 [0.1–16.4] pg/mL, p = 0.594).

## Discussion

This study provides evidence that sGFAP is elevated in ET-patients with late tremor onset in their early disease stage (ESLO) compared to HC. Furthermore, these patients experienced a markedly elevated sGFAP-increase over a five-years observational period when compared to ET-patients presenting with early disease onset or long disease durations (LSEO) and HC. Notably, in the ESLO-cohort but not the LSEO-cohort, higher baseline sGFAP-levels were associated with higher follow-up tremor severity.

When exploring the total ET-group, patients with high baseline sGFAP-levels were older at disease onset and baseline compared to patients with low baseline sGFAP-levels. Only in patients with high sGFAP-levels, annual sGFAP-increase correlated with annual tremor progression, which might reflect disease activity at least in these elderly, late-onset ET-patients.

Taken together, these in-vivo findings further support the notion of a neurodegenerative process in ET, particularly in later-onset disease. As suggested previously (Franthal et al. 2023), we believe that these factors occur primarily at an early stage of ET. The elevation of sGFAP, representing an astrocytic marker, may indicate astrocytic involvement in the suspected neurodegenerative process, whether due to primary degeneration or in a compensatory mechanism. Interestingly, the adult form of Alexander’s disease, which is caused by mutations in the GFAP gene, frequently exhibits cerebellar signs including tremor (Srivastava et al. 1993), and up to 45% of patients with autoimmune GFAP-astrocytopathy have tremor (Kimura et al. 2022). In this context, it appears to be of particular interest that annual sGFAP-increase correlates with annual increase of FTM subscores A and B, which rate objective motor symptom severity, but not subscore C, which assesses functional disability (Fahn et al. 1988). As stated above, this dynamic correlation between sGFAP and tremor severity found in a subgroup of our small sample might suggest sGFAP as a potential marker particularly for motor disease activity in essential tremor with late disease onset; however, this finding warrants further reproduction in larger, prospectively planned studies.

Considering our current findings together with previously reported elevated sNfL in early disease stages (Franthal et al. 2023) and microstructural cerebellar alterations, especially in cerebellar peduncles and dentate nuclei of ET-patients (Holtbernd and Shah 2021; van den Berg and Helmich 2021), we hypothesize that cerebellar decoupling (Madelein van der Stouwe et al. 2020) in consequence of a neurodegenerative process, happening at an early disease stage, might contribute to pathophysiology in late-onset ET. However, further research combining blood biomarkers and neuroimaging is needed to test this hypothesis. While head tremor has been associated to more pronounced cerebellar atrophy in ET-patients (Holtbernd and Shah 2021), sGFAP-levels did not differ between patients with and without head tremor in our cohort. However, the interpretation of this observation might possibly be limited by the small number of patients with head tremor in our population (n = 6 at baseline, n = 5 at follow-up).

While ESLO-patients were slightly older at baseline than LSEO-patients and HC, they also had a slightly higher BMI and lower proportion of females, probably leading to a leveling of these potential confounders (Abdelhak et al. 2022; Yalachkov et al. 2022; Gonzales et al. 2023).

In contrast to differentiating late- and early-onset patients, stratifying in ET-plus vs. pure-ET did not reveal differences in sGFAP-levels in our population. The ET-plus concept has been criticized as being solely based on subtle clinical findings of uncertain significance rather than pathophysiological considerations (Louis et al. 2019). While some authors suggest that ET-plus might merely represent a disease stage rather than a disease entity (Louis et al. 2021), others have shown that ET-plus-patients have a later disease onset than pure-ET-patients (Erro et al. 2022).

Our study has several strengths and limitations. This explorative pilot study is the first to report data on sGFAP in ET-patients. Another strength is the longitudinal design with low loss to follow-up. Moreover, our results in different subgroups substantiate each other. Limitations include small sample sizes and the overall quite high median age and disease duration of our ET-population. While most studies suggest ET-plus being more prevalent than pure-ET (Rajalingam et al. 2018; Erro et al. 2022), only 44.4% of patients were classified as ET-plus in our cohort. However, most of our patients were recruited before the publication of the MDS 2018 criteria for ET-plus, hence classification could only be performed retrospectively based on video recordings of the baseline examination. At the same time, slight resting tremor or subtle dystonia often are not caught on video tape, possibly leading to an erroneous classification of pure-ET and simultaneously under-classification of ET-plus. Therefore, we attribute the low percentage of ET-plus in our study cohort to the predominantly retrospective video-based classification of ET-patients in pure ET vs. ET-plus.

This study is the first to provide evidence based on serum biomarkers that late-onset ET might represent a distinct disease subtype. As a major limitation, our data did not allow to directly compare late-onset to early-onset patients [without the second stratification of disease duration, performed based on our previous findings (Franthal et al. 2023)], which would have led to differences in baseline age, hence confounding the results. Thus, we recognize that our results cannot be generalized to late-onset-ET in general, but rather must be restricted to the early disease stage of those patients. Our study cohort did also not allow for a further stratification into the consequential subtypes of “early-stage early-onset” (ESEO) and"late-stage late-onset” (LSLO). This is, on the one hand, due to the small sample size of the study. On the other hand, the sample was drawn from consecutive patients presenting in a tertiary movement disorders outpatient clinic. While ESEO-patients would generally only be mildly affected by insidious onset tremor, LSLO-patients might not be sent to an academic center for various reasons (insufficient longevity, other medical comorbidities in the foreground, frailty). Hence, both subgroups tend not to present to our tertiary movement disorders center. Consequently, our findings raise the need for larger, prospectively conducted studies, allowing to compare age-matched early vs. late-onset patients, and ESEO- and LSLO-patients should be actively recruited for future studies.

In conclusion, sGFAP is elevated in late-onset ET-patients in their early disease stages, and those patients also experience a higher sGFAP-increase over time. Higher sGFAP-increase might reflect tremor progression and hence disease activity, at least in elderly ET-patients with late disease onset.

## Data Availability

The data that support the findings of this study are available on request from the corresponding author.

## Declarations

### Standards of reporting

This observational study followed the Strengthening the Reporting of Observational Studies in Epidemiology (STROBE) checklist guidelines for case–control studies.
